# Specific Sirt1 Activator-mediated Improvement in Glucose Homeostasis Requires Sirt1-Independent Activation of AMPK

**DOI:** 10.1016/j.ebiom.2017.03.019

**Published:** 2017-03-14

**Authors:** Sung-Jun Park, Faiyaz Ahmad, Jee-Hyun Um, Alexandra L. Brown, Xihui Xu, Hyeog Kang, Hengming Ke, Xuesong Feng, James Ryall, Andrew Philp, Simon Schenk, Myung K. Kim, Vittorio Sartorelli, Jay H. Chung

**Affiliations:** a Laboratory of Obesity and Aging Research, Genetics and Developmental Biology Center, National Heart Lung and Blood Institute, National Institutes of Health, Bethesda, MD 20892, USA; b Translational Medicine Branch, National Heart Lung and Blood Institute, National Institutes of Health, Bethesda, MD 20892, USA; c Department of Biochemistry and Biophysics, The University of North Carolina, Chapel Hill, NC 27599, USA; d Laboratory of Muscle Stem Cells and Gene Regulation, National Institute of Arthritis and Musculoskeletal and Skin Diseases, National Institutes of Health, Bethesda, MD 20892, USA; e School of Sport, Exercise and Rehabilitation Sciences, University of Birmingham, Edgbaston, Birmingham, United Kingdom; f Department of Orthopedic Surgery, University of California San Diego, La Jolla, CA 92093, USA

**Keywords:** SRT1720, Phosphodiesterases, cAMP, AMPK, Sirt1, Epac, Type 2 diabetes, Obesity, Aging, Mitochondria

## Abstract

The specific Sirt1 activator SRT1720 increases mitochondrial function in skeletal muscle, presumably by activating Sirt1. However, Sirt1 gain of function does not increase mitochondrial function, which raises a question about the central role of Sirt1 in SRT1720 action. Moreover, it is believed that the metabolic effects of SRT1720 occur independently of AMP-activated protein kinase (AMPK), an important metabolic regulator that increases mitochondrial function. Here, we show that SRT1720 activates AMPK in a Sirt1-independent manner and SRT1720 activates AMPK by inhibiting a cAMP degrading phosphodiesterase (PDE) in a competitive manner. Inhibiting the cAMP effector protein Epac prevents SRT1720 from activating AMPK or Sirt1 in myotubes. Moreover, SRT1720 does not increase mitochondrial function or improve glucose tolerance in AMPKα2 knockout mice. Interestingly, weight loss induced by SRT1720 is not sufficient to improve glucose tolerance. Therefore, contrary to current belief, the metabolic effects produced by SRT1720 require AMPK, which can be activated independently of Sirt1.

## Highlights

• SRT1720 activates AMPK in a Sirt1-independent manner.
• SRT1720 activates AMPK by inhibiting cAMP phosphodiesterase.
• SRT1720-mediated improvement in glucose homeostasis requires AMPK.
• Weight loss due to SRT1720 is not sufficient for improved glucose homeostasis.

Obesity has become an epidemic and obesity-related diseases such as type 2 diabetes are on the rise. Therefore, discovering novel therapies for these diseases will have great public health impact. Sirt1 activating compounds such as SRT1720 protect against obesity and glucose intolerance, but the mechanism by which they confer these health benefits has been unclear. We discovered that SRT1720 activates energy sensor AMPK, independent of Sirt1, and increases mitochondrial function and glucose tolerance in an AMPK-dependent manner. SRT1720 activates AMPK by directly inhibiting cAMP phosphodiesterases, suggesting that cAMP phosphodiesterases may be potential drug targets for obesity-related diseases.

## 1 Introduction

Calorie restriction (CR) has been reported to extend the lifespan of many model organisms including rodents and possibly non-human primates (Colman et al., 2009, Guarente, 2000, McCay et al., 1935). It has been proposed that some of the positive health effects of CR are mediated, at least in part, by the Sir2 family of NAD^+^-dependent protein deacetylases (Guarente, 2000). Although increasing the expression levels of Sirt1, the mammalian orthologue of Sir2, does not extend lifespan, it appears to extend healthspan (Bordone and Guarente, 2005, Herranz et al., 2010), improve insulin sensitivity and protect against hepatic steatosis in transgenic mice (Banks et al., 2008, Bordone et al., 2007, Boutant et al., 2016, Pfluger et al., 2008). Therefore, Sirt1 may be a target for developing therapeutics to treat aging-related diseases.

Resveratrol, a polyphenol found in red wine and other plant-based foods, was the first of the Sirt1-activating compounds (STACs) to be reported and has been described as a CR-mimetic (Howitz et al., 2003). Resveratrol increased mitochondrial biogenesis, metabolic rate, insulin sensitivity and glucose tolerance and protected against premature death due to a high fat diet in mice (Baur et al., 2006, Lagouge et al., 2006). However, resveratrol was not Sirt1-specific (Borra et al., 2005, Kaeberlein et al., 2005) so a group of synthetic STACs, which are structurally unrelated to resveratrol, were subsequently developed. Synthetic STACs such as SRT1720 protected mice against obesity; increased mitochondrial biogenesis, energy metabolism and glucose tolerance (Feige et al., 2008, Milne et al., 2007); decreased hepatic lipid accumulation (Walker et al., 2010, Yamazaki et al., 2009) and extended the lifespan in mice (Minor et al., 2011b, Mitchell et al., 2014). Because weight loss can be related to all of these benefits, it was not known whether they are all simply due to the weight loss induced by SRT1720.

There is a complex interrelationship between Sirt1 and AMP-activated kinase (AMPK), which regulates energy balance by stimulating pathways involved in mitochondrial biogenesis, energy production and glucose uptake and suppressing glucose production (Hardie, 2013). The activities of Sirt1 and AMPK are mutually interdependent: Sirt1-mediated deacetylation of LKB1, an upstream kinase for AMPK, translocates it to the cytoplasm facilitating interaction with and activation of AMPK (Lan et al., 2008), and AMPK can promote Sirt1-dependent pathways by diverse mechanisms, including the disruption of Sirt1 interaction with DBC1, its inhibitor (Canto et al., 2009, Chang et al., 2015, Fulco et al., 2008, Nin et al., 2012, Um et al., 2010).

SRT1720 is now the most widely used Sirt1-specific activator. However, the question of whether SRT1720 exerts its metabolic effects by directly activating Sirt1 still remains unanswered (Dai et al., 2010, Huber et al., 2010, Pacholec et al., 2010). A number of groups have shown that STACs can allosterically activate Sirt1 against substrates with a fluorophore tag, but not native substrates (Borra et al., 2005, Kaeberlein et al., 2005). More recently, it was reported that STACs can activate Sirt1 against substrates with a bulky hydrophobic amino acid (tyrosine, tryptophan or phenyalanine) at positions + 1 or + 6 in the absence of a fluorophore tag (Dai et al., 2015, Dai et al., 2010, Hubbard et al., 2013). This was then contradicted by Cao et al. who found that even with a bulky hydrophobic amino acid at + 1 or + 6, there was no activation without a fluorophore tag (Cao et al., 2015). Nevertheless, the metabolic effects of SRT1720 have been reported to require Sirt1 (Feige et al., 2008, Minor et al., 2011b, Mitchell et al., 2014).

If STACs such as resveratrol and SRT1720 increase mitochondrial function and protect against obesity by directly activating Sirt1, it is reasonable to predict that transgenic mice with increased expression of Sirt1 from its native promoter (gain of function) will have increased mitochondrial function and be protected against obesity (Banks et al., 2008, Boutant et al., 2016, Pfluger et al., 2008). However, these transgenic mice did not have increased mitochondrial function and were not protected against obesity, which are two of the salient effects of the SRT1720 treatment. The simplest explanation of these findings is that increased Sirt1 activity is not the central driving force in SRT1720 action.

Here, we examine the central mechanism behind the metabolic effects of SRT1720 in a diet-induced obesity model. We find that contrary to the prevailing belief, SRT1720 activates AMPK and this occurs in a Sirt1-independent manner. Moreover, SRT1720 activates AMPK by activating the cAMP pathway and confers anti-diabetic effects in an AMPK-dependent manner. Improvement in glucose tolerance is not simply due to the weight loss induced by SRT1720 as AMPK-deficient mice lose weight on SRT1720 without improving glucose tolerance. Since the metabolic effects of SRT1720 have been shown to require Sirt1 (Feige et al., 2008, Minor et al., 2011b, Mitchell et al., 2014), we conclude that Sirt1-independent activation of AMPK is required for SRT1720 to improve glucose homeostasis.

## 2 Material and methods

### 2.1 Mice

**Fig. 1:**
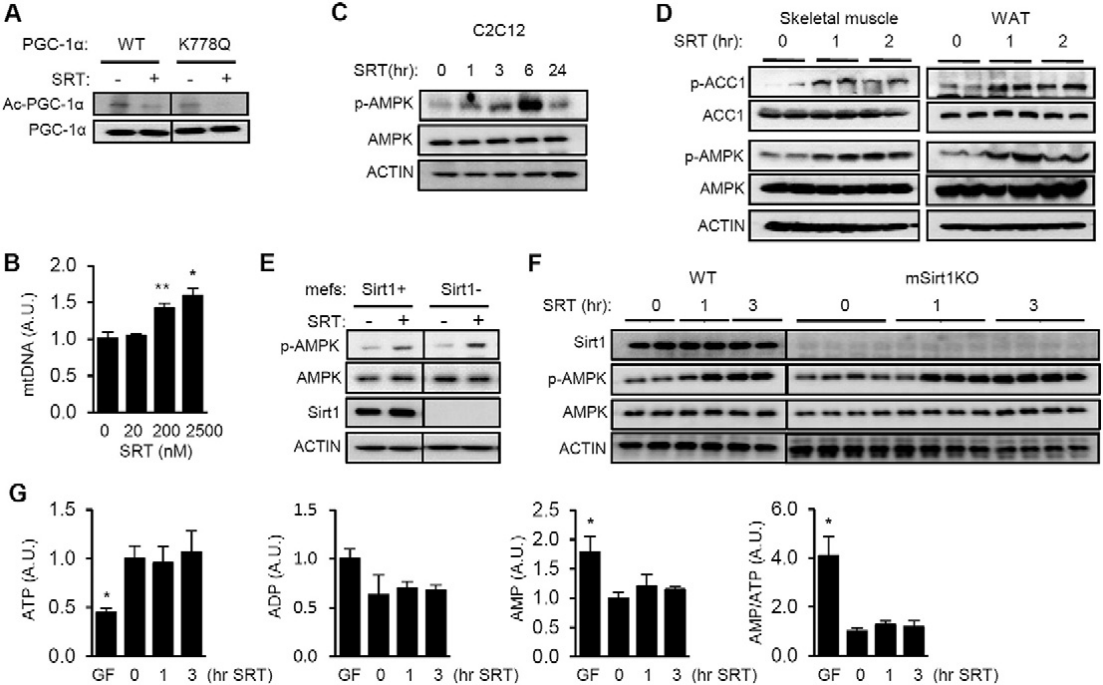
SRT1720 activates AMPK in a Sirt1-independent manner. (A) C2C12 myotubes transfected with WT or the K778Q mutant of PGC-1α were treated with SRT1720 (2.5 μM). The acetylation level of PGC-1α was measured by immunoprecipitating with PGC-1α and immunoblotting with antibody specific for anti-acetylated lysine. (B) Relative amounts of mitochondrial DNA (mtDNA) in C2C12 myotubes after treatment with SRT1720 for 3 days. Genomic DNA was used as the internal control. All values are given as mean ± s.e.m. *p < 0.05; **p < 0.01. (C) AMPK activity (Thr-172 phosphorylation, p-AMPK) in C2C12 after SRT1720 (2.5–5 μM) for the indicated times. (D) SRT1720 (10 mg/kg) was injected (*i.p.*) into mice and the phosphorylation status of AMPK (p-AMPK) and AMPK substrate acetyl-CoA carboxylase (p-ACC) were visualized by immunoblotting in skeletal muscle and white adipose tissue (WAT). (E) AMPK activity (Thr-172 phosphorylation, p-AMPK) in Sirt1 −/− mefs (Sirt1 −) and Sirt1 −/− mefs with restored Sirt1 (Sirt1 +) after SRT1720 (2.5 μM) treatment. (F) AMPK activation (p-AMPK) in skeletal muscle isolated from WT and muscle specific Sirt1 KO mice after i.p injection with SRT1720 (30 mg/kg). (G) Intracellular ATP, ADP, AMP levels and AMP/ATP ratio in C2C12 cells incubated with SRT1720 (2.5 μM) or glucose-free media for the indicated time periods. All values are given as mean ± s.e.m. *p < 0.05.

All experiments were approved by the NHLBI ACUC (Animal Care and Use Committee). Wild-type C57BL/6J mice were originally purchased from Jackson Laboratory. Muscle-specific KO of Sirt1 was accomplished by crossing Sirt1^fl/fl^ mice with Pax7-Cre mice as described previously (Ryall et al., 2015). AMPK α2 −/− (Viollet et al., 2003) mice were backcrossed to C57BL/6J for at least six generation before this study. For the experiment shown in Fig. 1D, Fig. 3, Fig. 4, Fig. 5 mo old C57BL/6J mice that were fed Lab Diet 5021 were injected *i.p.* with 10 mg/kg SRT1720. For the experiment shown in Fig. 1F, WT and Muscle-specific Sirt1 KO mice that were fed regular chow (NIH-31) were injected *i.p.* with 30 mg/kg SRT1720 when they were 3–5 mo old. For high fat diet (HFD) studies with SRT1720 in WT and/or AMPKα2 KO mice, the mice were dosed once daily by oral gavage with or without 100 mg/kg/d SRT1720 in 10% PEG400 in saline (10 μl/g body weight) for up to 10 weeks. The dose was increased to 300 mg/kg/d from 11 weeks. Body weight and caloric intake were monitored throughout the experiments. Locomotor activity of mice was measured by photobeam breaks by using the Opto-Varimex-4 (Columbus Instruments). Mice were housed with a 12 h light-dark cycle (light on 6 am-6 pm) with free access to food and water.

### 2.2 Insulin Tolerance Test (ITT) and Glucose Tolerance Test (GTT)

Plasma glucose was measured by using a glucometer (Ascensia). For the glucose tolerance test and insulin tolerance test, mice were fasted for 16 h, and 1 mg/g glucose or 0.75 mIU/g insulin were injected intraperitoneally (*i.p*.). Blood glucose was measured at 0, 20, 40, 60 and 80 min after injection.

### 2.3 Cell Culture

C2C12 cells and Hela cells were maintained in complete Dulbecco's modified Eagle's medium (DMEM) medium supplemented with 10% fetal bovine serum (FBS) (growth media) and 100 μg/ml penicillin and streptomycin. C2C12 cells were used before passage 25. When C2C12 cells were nearly confluent (70%–80% confluency), they were induced to differentiate into myotubes by replacing the growth media with differentiation media (DMEM supplemented with 2% horse serum).

### 2.4 Whole-cell Lysate Preparation

Cell pellets were lysed on ice for 20 min in RIPA buffer (50 mM Tris-HCl, pH 7.4, 0.15 M NaCl, 1.0 mM EDTA, 1% NP-40, 0.25% sodium deoxycholate) freshly supplemented with phosphatase and protease inhibitors (Millipore). Lysates were clarified by centrifugation at 14,000 rpm for 10 min. Proteins were quantified by using the Coomassie plus protein assay (Thermo Fisher scientific) and lysates were either used immediately or stored at − 80 °C.

### 2.5 Mitochondrial DNA (mtDNA) Quantification by Quantitative Real-Time PCR

Relative amounts of nuclear DNA and mtDNA were determined by quantitative Real-Time PCR. The ratio of mtDNA to nuclear DNA reflects the tissue concentration of mitochondria per cell. Skeletal muscle was homogenized and digested with Proteinase K overnight in a lysis buffer for DNA extraction by DNeasy blood and tissue kit (QIAGEN). Quantitative PCR was performed using each primers (mtDNA specific PCR, forward 5′-CCGCAAGGGAAAGATGAAAGA-3′, reverse 5′-TCGTTTGGTTTCGGGGTTTC-3′; and nuclear DNA specific PCR, forward 5′-GCCAGCCTCTCCTGATTTTAGTGT-3′, reverse 5′-GGGAACACAAAAGACCTCTTCTGG-3′) and Power SYBR Green PCR master mix (Applied Biosystems) in a 7900T Real-Time PCR system (Applied Biosystems). The PCR reactions consisted of 10 μl Power SYBR Green PCR Master mix (2 ×), 7 μl RNAse free water, 1 μl 300 nM primer mix and 2 μl cDNA, to a total volume of 20 μl. Three technical replicates were performed for each sample. The cycling conditions were 15 min at 95^o^ C, followed by 50 to 60 cycles of 15 s at 95^o^ C, 20 s at 58^o^ C and 20 s at 72^o^ C.

### 2.6 Tissue Lysate Preparation

Frozen tissues were ground and homogenized in a mortar and pestle under liquid nitrogen. Samples were then incubated in RIPA buffer (50 mM Tris-HCl, pH 7.4, 0.15 M NaCl, 1.0 mM EDTA, 1% NP-40, 0.25% sodium deoxycholate) freshly supplemented with phosphatase and protease inhibitors (Millipore). In order to break the tissue up further and to shear DNA, lysates were sonicated briefly (5 s, 140 watt, setting 7 using on Ultrasonics W-385 Sonicator (Heat Systems), which were then vortexed every 30 min. The homogenates were incubated at 4 °C for 2 h and then centrifuged (13,000 rpm) for 15 min at 4 °C, and supernatants were collected.

### 2.7 SRT1720

SRT1720 was synthesized according to a procedure described previously (Milne et al., 2007) and was confirmed by NMR and mass spectrometry. The final product was purified by high-performance liquid chromatography. We used SRT1720 synthesized and purified by Natland International Corporation (purity of > 95%).

### 2.8 siRNA

Human Epac1 siRNA and PKAc (α) siRNA were purchased from Dharmacon. Control siRNA was also from Dharmacon. Epac1 and PKAc (α) were knocked down by transfecting Hela cells with siRNA by using Lipofectamine 2000 according to the manufacturer's protocol. Four days after transfection, cells were harvested and lysed.

### 2.9 Cyclic AMP Measurement

The cyclic AMP complete enzyme immunoassay kit from Assay Designs was used as directed by the manufacturer.

### 2.10 ATP Measurement

The Staybrite highly stable ATP bioluminescence assay kit from Biovision was used as directed by the manufacturer.

### 2.11 ADP and AMP Measurement

Intracellular nucleotides were extracted and measured by HPLC as described before (Shryock et al., 1986). Briefly, ice-cold 5% perchloric acid (PCA) was added into the cell pellet followed by briefly sonicating twice. Next, the samples were spun at 10,000 rpm for 10 min at 4 °C. The supernatant was collected and stored at − 80 °C for nucleotides measurement by the Agilent HPLC 1100 system. The pellet was lysed by RIPA buffer for quantifying protein levels and normalizing nucleotide release.

### 2.12 Ca^2 +^ Signal Measurement

C2C12 myoblasts were seeded on a 96 well plate (Perkin Elmer). After differentiation, they were preincubated with 20 μM U73122 for 1 h. Ca^2^^+^ release was measured using the fluorescent calcium indicator Fluo-4AM (Molecular Probes) according to the manufacturer's suggestions. Ca^2^^+^ increases are reported as ΔF/F ((F-Fbasal)/Fbasal), where F indicates fluorescence.

### 2.13 Transfection of Full-length and Catalytic Domain PDE4

Expression vectors for full-length PDE4 and the catalytic domain of PDE4 were constructed by inserting the cDNA encoding His-tagged full-length PDE4D7 or the catalytic domain of PDE4D (Burgin et al., 2010) into the mammalian expression vector pCDNA3. Subconfluent C2C12 myocytes (80%) were transfected by using the Lipofectamine 2000 transfection reagent with expression vectors for full-length PDE4 or the catalytic domain of PDE4. Myocytes were then differentiated into myotubes according to the standard protocol by using DMEM containing 2% horse serum.

### 2.14 Immunoblotting

Cells were lysed in RIPA buffer and subjected to immunoblotting. For tissue extraction, samples were pulverized in liquid nitrogen and homogenized in the lysis buffer. The following antibodies were used: AMPK (Cell Signaling Technology), p-AMPK (T172) (Cell Signaling Technology), p-ACC, which recognizes phosphorylated Ser 79 in ACC1 (Cell Signaling Technology), total ACC (Cell Signaling Technology), Rap1 (Cell Signaling Technology), EPAC1 (Cell Signaling Technology), PKAcα (Cell Signaling Technology), RyR2 (Abcam), p-RyR2 (Abcam), Sirt1 (Upstate Biotechology), V5 (Invitrogen) and Actin (Santa Cruz). PGC-1α acetylation was visualized by immunoprecipitation from the cell extract (500 μg) using PGC-1α antibody (Santa Cruz) followed by immunoblotting with antibody specific for acetylated lysine (Cell Signaling Technology) or for PGC-1α.

### 2.15 Real-time PCR

Frozen skeletal muscles were ground and homogenized in a mortar and pestle under liquid nitrogen. Total RNA was extracted using TRIzol (Invitrogen) according to the manufacturer's protocol. Complementary DNA (cDNA) was synthesized from 2 μg of DNA-free total RNA by using the High Capacity cDNA Archive kit (Applied Biosystems). 10 μl of RNA (2 μg) was mixed with 10 μl of reverse transcriptase master mix (2 ×), which contained 2 μl of 10 × RT buffer, 2 μl of 10 × Random Primers, 0.8 μl of 25 × dNTP mix, 1 μl of MultiScribe RT (50 U/ul) and 4.2 μl of RNAse free water, giving a final volume of 20 μl. The reaction mixtures were incubated at 25 °C for 10 min and 37 °C for 120 min. Reverse transcription products were diluted 1:3 in nuclease-free water, and 1 μl was used for RT-PCR with the Taqman core reagents RT-PCR kit (Applied Biosystems) in combination with the 7900T Real-Time PCR System (Applied Biosystems). The RT-PCR was performed for 40 cycles at the following cycling condition: 5 °C for 10 min initial denaturation, then 40 cycles at 95 °C denaturation, 60 °C anneal/extension for 15 s and 1 min at each temperature, respectively. 18S RNA was used as the internal standard for all mRNA. The following primers (Applied Biosystems) were used in these studies: PGC-1α, Mm00447183_m1; PGC-1β, Mm00504720_m1; MCAD, Mm00431611_m1; NRF1, Mm00447996_m1; ERRα, Mm00433143_m1; Tfam, Mm00447485_m1; Euk 18SrRNA, 4333760F.

### 2.16 Rap1 Pull Down Assay

pGEX Ral GDS-RA, an expression vector for GST-RalGDS-RBD (van Triest et al., 2001), was transformed into *E. coli* (strain BL21). Protein production was initiated by addition of isopropyl-β-D-thiogalactopyranoside (IPTG) to the culture. The fusion protein was affinity purified on a glutathione sepharose 4B column (Amersham Bioscience) from the supernatant of bacteria lysed by sonication. GST-RalGDS-RBD pre-coupled to a glutathione sepharose 4B column was added to the cell lysates and incubated at 4 °C for 60–180 min with slight agitation. Beads were washed four times in lysis buffer and subjected to immunoblotting.

### 2.17 PDE Assay

PDE activity was measured by modification of a previously published method (Ahmad et al., 2009) by using 10 nM [^3^H]cAMP (45,000 cpm) or [^3^H]cGMP as substrates. < 10%–15% of the substrates were hydrolyzed during the PDE reaction. Portions of solubilized cell lysates were assayed for PDE activity by incubation with SRT1720 (0–100 μM) or with specific PDE inhibitors. Recombinant PDE1 (10 ng) activity was assayed by using 4 μg/ml calmodulin and 0.8 mM Ca^2 +^ together with [^3^H]cAMP as substrate in the reaction mixture. Recombinant PDE2 (15 ng) activity was assayed in the presence of 1 μM cGMP, which activated it by ∼ 3 fold. Activities of recombinant PDE3 (1 ng) and PDE4 (1 ng) were assayed by incubation in a reaction mixture containing 1 mg/ml BSA, with [^3^H]cAMP. Recombinant PDE5 (150 ng) activity was assayed using [^3^H]cGMP as substrate.

### 2.18 Citrate Synthase Activity Assay

The Citrate synthase activity assay kit from Cayman chemical was used as directed by the manufacturer.

### 2.19 Statistical Analysis

Comparisons between the treatment groups were analyzed by two-tailed Student's *t*-test. Results are expressed as the mean ± s.e.m. Significance was accepted at p < 0.05.

## 3 Results

### 3.1 SRT1720 Activates AMPK in a Sirt1-independent Manner

Hubbard et al. found that in order for STACs to activate Sirt1 *in vitro*, the substrate needed to have a bulky hydrophobic residue at position + 1 or + 6 (Hubbard et al., 2013). This observation prompted us to determine if this condition also applies to SRT1720 action *in vivo*. Peroxisome proliferator-activated receptor gamma coactivator-1α (PGC-1α) (Puigserver et al., 1998), the master regulator of mitochondrial biogenesis and function (Jager et al., 2007), is a critical Sirt1 substrate (Nemoto et al., 2005, Rodgers et al., 2005). Since out of 13 lysines in PGC-1α, only K778 has a bulky hydrophobic amino acid at position + 1 or + 6, we tested whether mutating PGC-1α K778 would prevent SRT1720 activation of Sirt1-mediated PGC-1α deacetylation in C2C12 myotubes. As shown in Fig. 1A, SRT1720 stimulated deacetylation of PGC-1α not only at the lysine(s) in WT PGC-1α but also at the remaining lysine(s) lacking the nearby bulky hydrophobic amino acids in the K778Q mutant PGC-1α. This suggests that at least for PGC-1α, SRT1720 activates Sirt1 independently of the bulky hydrophobic residues *in vivo* and/or that SRT1720 can activate Sirt1 indirectly.

Since Sirt1 and AMPK have a reciprocal relationship, we considered the possibility that some of the metabolic effects of SRT1720 may be a consequence of AMPK activation. However, it is the prevailing view that SRT1720 does not activate AMPK acutely in C2C12 myotubes and skeletal muscle. Feige et al. reported that SRT1720 does not activate AMPK in C2C12 myotubes (Feige et al., 2008), but the concentration of SRT1720 these authors used (0.01–0.05 μM) was significantly lower than the concentration used by others (1–10 μM) (Funk et al., 2010, Smith et al., 2009, Svensson et al., 2015) and the serum concentration of SRT1720 in mice treated with SRT1720 (~ 1 μM) (Milne et al., 2007). Therefore, it is possible that SRT1720 did not activate AMPK in C2C12 myotubes because the concentration of SRT1720 that was previously used *in vitro* (Feige et al., 2008) was too low. To test this possibility, we first determined the concentration of SRT1720 required to increase mitochondrial biogenesis in C2C12 myotubes (Fig. 1B). We found that SRT1720 significantly increases mitochondrial biogenesis at 0.2–2.5 μM concentration. To investigate the potential effect of SRT1720 on AMPK activity, we visualized phosphorylation of Thr172 of AMPK (p-AMPK), a marker of AMPK activity, after 1–24 h treatment with 2.5–5 μM SRT1720 in C2C12 myotubes. As shown in Fig. 1C, AMPK activation occurred 1–6 h after treatment but had subsided by 24 h. Svensson et al. (2015)) also reported that SRT1720 does not activate AMPK in C2C12 myotubes. However, Svensson et al. visualized p-AMPK 24 h after treatment and therefore may have missed earlier activation. Furthermore, injection of SRT1720 (10 mg/kg) increased AMPK activity in skeletal muscle and white adipose tissue (WAT) as evidenced by p-AMPK and specific phosphorylation of the AMPK substrate acetyl-CoA carboxylase (p-ACC, S79/221) (Fig. 1D). Taken together, these findings indicate that SRT1720 can activate AMPK.

To determine the role of Sirt1 in SRT1720 action on AMPK, we visualized AMPK activity in Sirt1 KO mouse embryonic fibroblasts (mefs) in which Sirt1 was restored (Sirt1 +) by stable transfection or not (Sirt1-) (Fig. 1E). SRT1720 activated AMPK in Sirt1- mefs as well as it did in Sirt1 + mefs. To confirm that SRT1720 activates AMPK in a Sirt1-independent manner *in vivo*, we initially considered using WT and muscle-specific Sirt1 KO mice that have been fed high fat diet (HFD), but we found that at baseline (prior to SRT1720 treatment), tissues from muscle-specific Sirt1 KO on HFD already showed signs of metabolic stress such as low ATP levels, presumably because of insufficient fat oxidation (data not shown). Comparisons of their response to SRT1720 would be difficult to interpret if their baseline states are different to begin with. Therefore, we injected SRT1720 into 3–5 mo old WT and muscle-specific Sirt1 KO mice that have been fed a regular chow and therefore did not have significant metabolic stress at baseline. As shown in Fig. 1F, we found that injection of SRT1720 activated AMPK equally in WT and muscle-specific Sirt1 KO muscle. To determine if SRT1720 activated AMPK by inhibiting intracellular ATP production, we measured ATP concentration after SRT1720 treatment, using glucose- free media as a positive control. As shown in Fig. 1G, glucose-free media decreased ATP by approximately 60%, but SRT1720 did not change the concentrations of ATP, ADP or AMP (Fig. 1G). Taken together, these findings indicate that SRT1720 can activate AMPK in a Sirt1- and ATP/AMP-independent manner.

### 3.2 SRT1720 is Competitive Inhibitor of cAMP-hydrolyzing Phosphodiesterases (PDEs)

**Fig. 2:**
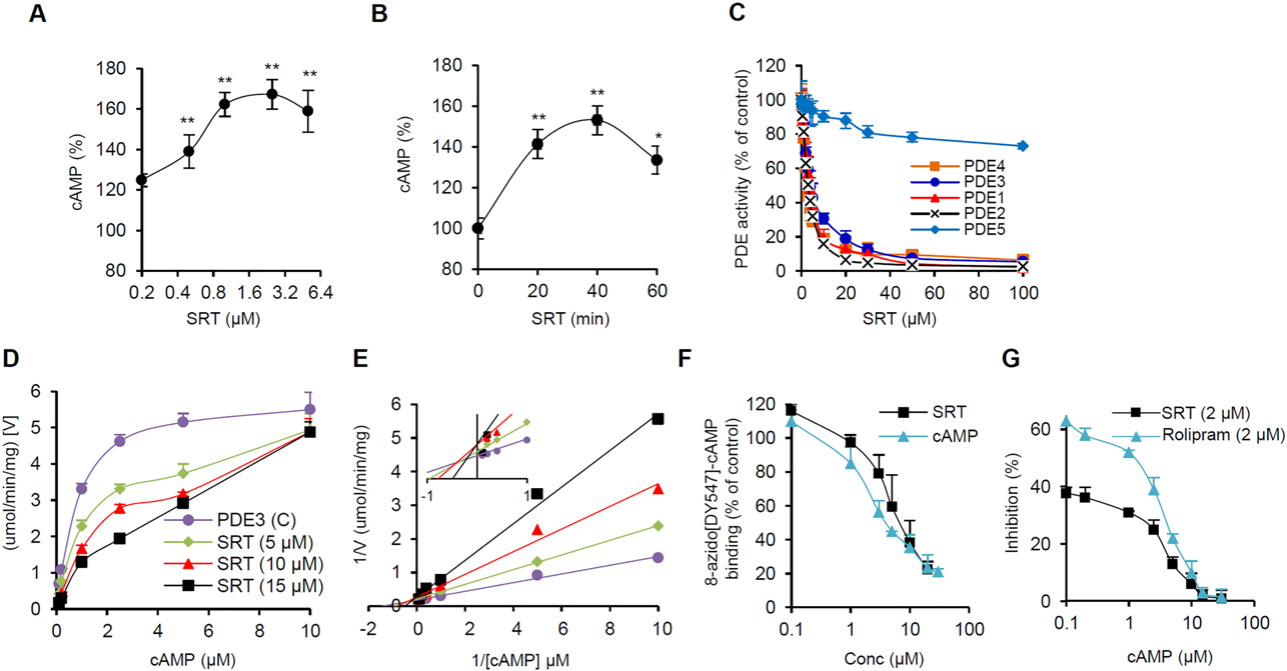
SRT1720 inhibits cAMP-specific phosphodiesterases by competing with cAMP. (A) Cyclic AMP levels in C2C12 myotubes were measured 30 min after treatment with the indicated concentrations of SRT1720. All values are given as mean ± s.e.m. *p < 0.05; **, p < 0.01. (B) Cyclic AMP levels in C2C12 myotubes were measured at the indicated times after treatment with 2.5 μM SRT1720. All values are given as mean ± s.e.m. *p < 0.05; **, p < 0.01. (C) The inhibitory effect of SRT1720 on recombinant PDE1–5 is shown. All values are given as mean ± s.e.m. (D) Velocity of recombinant PDE3 activity as a function of cAMP and SRT1720 concentration. All values are given as mean ± s.e.m. (E) Lineweaver-Burk plot of panel (D). (F) Recombinant PDE3 was photoaffinity-labeled with the fluorescent cAMP analog 8-azido-[DY-547]-cAMP in the presence of increasing concentrations of SRT1720 or cAMP. Quantification of 8-azido-[DY-547]-cAMP bound to PDE3 is shown. All values are given as mean ± s.e.m. (G) Inhibitory effects of SRT1720 (2 μM) and Rolipram (2 μM) on recombinant PDE4 activity as a function of cAMP concentration is shown. All values are given as mean ± s.e.m.

Since SRT1720 does not directly activate AMPK (data not shown), we searched for an indirect pathway that can activate AMPK. Pacholec et al. reported that SRT1720 inhibits recombinant cAMP phosphodiesterases (PDEs), which degrade cAMP, *in vitro* (Pacholec et al., 2010). Furthermore, resveratrol can activate AMPK by inhibiting cAMP phosphodiesterases (PDEs) (Park et al., 2012). To investigate the potential SRT1720-PDE link, we measured cAMP levels in C2C12 myotubes after treatment with SRT1720. As shown in Fig. 2A, the cAMP level increases by > 20% at 0.2 μM concentration and reaches maximum (> 60% increase) at 1–2 μM concentration. The peak cAMP level is reached approximately 40 min after treatment (Fig. 2B). We then measured the effect of SRT1720 on the activity of recombinant PDEs1-5. SRT1720 inhibited recombinant PDEs 1, 2, 3 and 4 but not PDE5, which hydrolyzes only cGMP (Fig. 2C). To gain a better understanding of how SRT1720 inhibits PDEs, we measured the effect of cAMP and SRT1720 on the kinetics of recombinant PDE3 activity. As shown in Fig. 2D, at high concentrations of cAMP, the inhibitory effect of SRT1720 on PDE3 disappeared, suggesting that SRT1720 competes with cAMP. A Lineweaver-Burk plot also indicated that SRT1720 and cAMP compete with each other (Fig. 2E). To directly demonstrate that SRT1720 competes with cAMP, we incubated PDE3 with the fluorescent cAMP analog 8-azido[DY547]-cAMP, which cross-links to its binding site, with increasing concentrations of either SRT1720 or cAMP. As shown in Fig. 2F, both SRT1720 and cAMP competed with 8-azido[DY547]-cAMP for the binding site in PDE. As seen in Fig. 2G, SRT1720 is approximately 30% weaker as a PDE4 inhibitor compared to rolipram, a well-known PDE4 inhibitor, and their inhibitory activity decreases in the presence of higher cAMP concentration in the PDE inhibition assay (Fig. 2G).

### 3.3 cAMP Effector Epac1 is Important for SRT1720-mediated Activation of AMPK

**Fig. 3:**
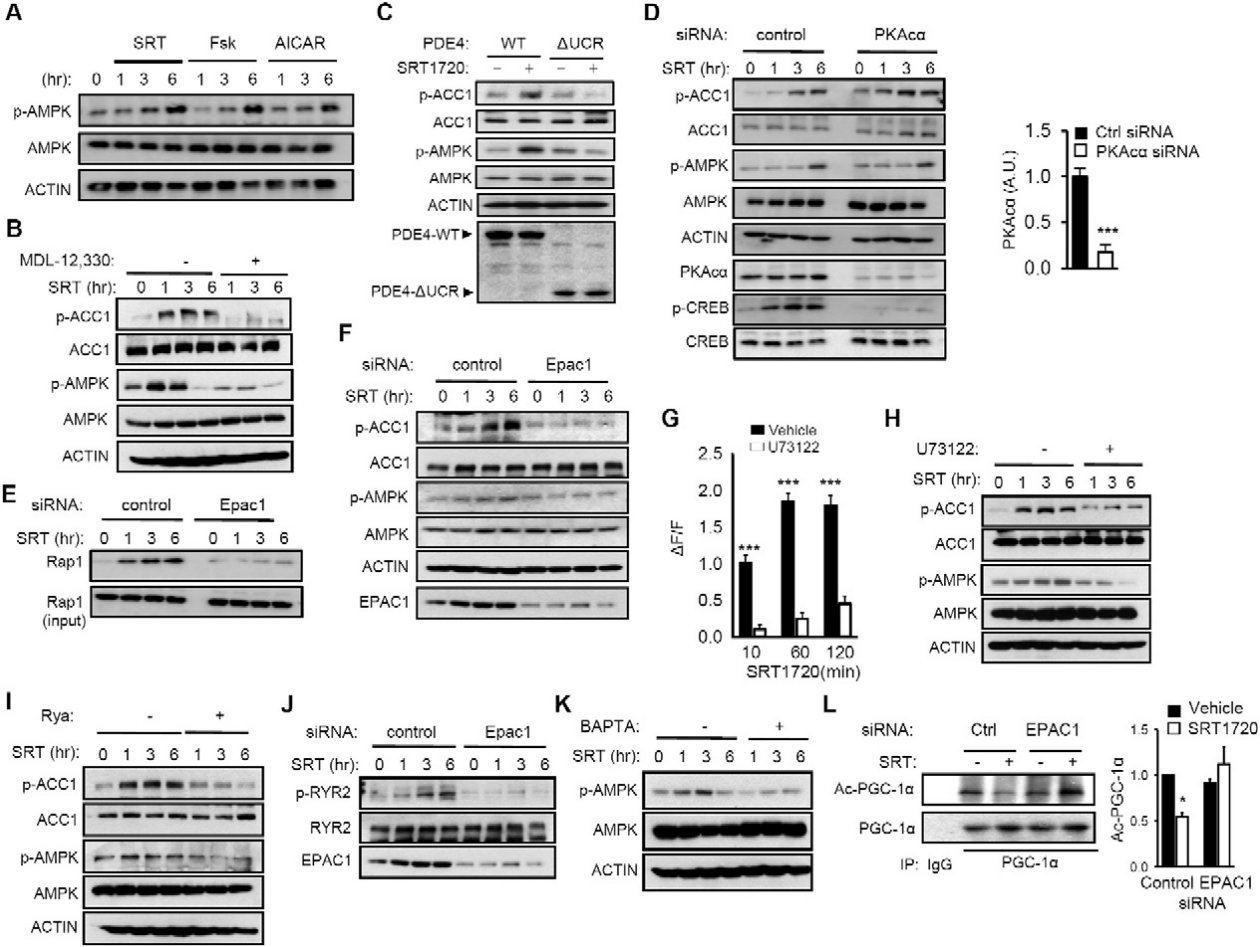
SRT1720 activates AMPK *via* the cAMP-Epac1 pathway. (A) C2C12 myotubes were treated with SRT1720 (2.5 μM) or Fsk (25 μM) or AICAR (200 μM), and p-AMPK was visualized by immunoblotting. (B) SRT1720-induced activation of AMPK requires cAMP. C2C12 myotubes were treated with SRT1720 (2 μM) for varying durations in the presence of the adenylyl cyclase inhibitor MDL-12,330. The phosphorylation status of ACC and AMPK were visualized by immunoblotting with phospho-specific antibodies. (C) Phosphorylation of ACC and AMPK in C2C12 myotubes overexpressing either His-tagged PDE4-WT or PDE4-ΔUCR after they were treated with SRT1720 (2.5 μM) for 3 h. The levels of His-tagged PDE4 were visualized by immunoblotting with anti-His antibody (bottom). (D) SRT1720-mediated activation of AMPK in the presence of PKAcα siRNA. AMPK activity was visualized by immunoblotting with antibody specific for p-ACC and p-AMPK in HeLa cells. Quantification of PKAcα expression level is shown on the right. (E) SRT1720 increases Epac activity. GTP-bound Rap1 was pulled-down using immobilized ras binding domain of RalGDS in the presence or absence of Epac1 siRNA. (F) SRT1720-mediated activation of AMPK in the presence of Epac1 siRNA. AMPK activity was visualized by immunoblotting with antibody specific for p-ACC and p-AMPK in HeLa cells. (G) SRT1720 increases cytosolic Ca^2 +^ in a PLC-dependent manner. The change in cytosolic Ca^2 +^-induced fluorescence normalized by the baseline fluorescence (ΔF/F) in C2C12 myotubes that were treated with SRT1720 in the presence or absence of the PLC inhibitor U73122 are shown. All values are given as mean ± s.e.m. ***p < 0.001. (H) PLC activity is required for SRT1720 to induce phosphorylation of ACC and AMPK. C2C12 myotubes were treated with SRT1720 in the presence or absence of U73122, and p-ACC and p-AMPK were visualized by immunoblotting. (I) Blocking Ryr Ca^2 +^ channel with ryanodine inhibits SRT1720-mediated phosphorylation of ACC and AMPK. C2C12 myotubes were treated with SRT1720 in the presence or absence of ryanodine and p-ACC and p-AMPK were visualized by immunoblotting. (J) SRT1720 increases phosphorylation of ER/SR Ca^2 +^ channel protein Ryr2 in an Epac1-depenent manner. After transfection with Epac1-specific siRNA, SRT1720-induced phosphorylation of S2815 of Ryr2 was visualized by immunoblotting. (K) The phosphorylation status of AMPK in C2C12 myotubes that were treated with SRT1720 in the presence of the Ca^2 +^ chelator BAPTA. (L) Epac1 siRNA blocks SRT1720 from activating Sirt1. The acetylated state of PGC-1α (Ac-PGC-1α) was quantified by scanning densitometry after immunoprecipitating PGC-1α and immunoblotting with antibody specific for anti-acetylated lysine (n = 3). Results are expressed as the mean ± s.e.m. *p < 0.05.

To demonstrate that SRT1720-induced production of cAMP is involved in AMPK activation, we compared treatment of C2C12 myotubes with SRT1720 to treatment with forskolin, an adenylate cyclase activator, as well as to treatment with the AMPK activator 5-Aminoimidazole-4-carboxamide ribonucleotide (AICAR). As shown in Fig. 3A, they all activated AMPK similarly. To confirm that SRT1720 activated AMPK in a cAMP-dependent manner, we blocked cAMP production in C2C12 myotubes with the adenylate cyclase inhibitor MDL-12,330 and found that it prevented SRT1720 from activating AMPK (Fig. 3B). Although SRT1720 inhibits PDEs *in vitro*, genetic evidence that PDE inhibition correlates with AMPK activation *in vivo* would strengthen our hypothesis that SRT1720 activates AMPK by inhibiting PDEs. PDE4 is the most abundant PDE in skeletal muscle (Park et al., 2012), but the PDE4 family is composed of four genes (A-D) with partially redundant functions, making it difficult to use knockout mice. Therefore, we sought an alternative strategy to demonstrate the role of PDE4 in SRT1720 action. The upstream conserved regions (UCRs) of PDE4, which are outside of the catalytic domain, increase the sensitivity of PDE4 to competitive inhibitors (Burgin et al., 2010). Therefore, PDE4 missing the UCR (ΔUCR) is relatively resistant to competitive inhibitors. If SRT1720 activates AMPK by competitively inhibiting PDE4, AMPK activity in C2C12 myotubes over-expressing the ΔUCR PDE4 should be relatively resistant to SRT1720 compared to that in C2C12 myotubes over-expressing WT PDE4. Indeed, the response of AMPK to SRT1720 was significantly weaker in C2C12 myotubes expressing the ΔUCR mutant than in C2C12 myotubes overexpressing WT PDE4 (Fig. 3C), supporting the notion that SRT1720 activates AMPK in a PDE4-dependent manner in C2C12 myotubes.

Downstream effects of cAMP signaling are largely mediated by two effectors: protein kinase A (PKA) (Taylor et al., 2013) and cAMP-regulated guanine nucleotide exchange factors (Epac) (de Rooij et al., 1998, Kawasaki et al., 1998). We used siRNA specific for PKAcα, the catalytic subunit of PKA, to knock down PKA activity before treatment with SRT1720. As shown in Fig. 3D, PKAcα siRNA did not significantly inhibit AMPK activation. Epac functions as a guanine nucleotide exchange factor for Rap1 and Rap2, which cycle between an inactive GDP-bound state and an active GTP-bound state. Epac proteins increase the fraction of Rap that is GTP-bound, which can be detected by a pull-down assay using the immobilized Ras association (RA) domain of ral guanine nucleotide dissociation stimulator (RalGDS) (van Triest et al., 2001). The pull-down assay showed that the levels of GTP-bound Rap1 in C2C12 myotubes was increased by SRT1720, indicating that SRT1720 increased Epac activity (Fig. 3E). To determine if Epac is important for SRT1720 to activate AMPK, we treated cells with Epac1 siRNA prior to SRT1720 treatment. We found that Epac1 siRNA blocked SRT1720 from activating AMPK (Fig. 3F). Thus, SRT1720 increases Epac1 activity and Epac1 activity is important for SRT1720 to activate AMPK.

Activation of Epac increases cytosolic Ca^2 +^ in a phospholipase Cε (PLCε)-dependent manner (Oestreich et al., 2009, Oestreich et al., 2007, Schmidt et al., 2001) and activates the Ca^+ 2^/calmodulin-dependent protein kinase kinase-β (CamKKβ)-AMPK pathway (Park et al., 2012). We investigated whether SRT1720 can activate AMPK in C2C12 myotubes by increasing the cytosolic Ca^2 +^ levels in a PLC-dependent manner. We found that SRT1720 increased cytosolic Ca^2 +^ levels but the PLC inhibitor U73122 not only blocked SRT1720`s ability to increase cytosolic Ca^2 +^ levels (Fig. 3G) but also its ability to activate AMPK (Fig. 3H). With Epac activation, the Ryr2 Ca^2 +^-release channel in endoplasmic reticulum/sarcoplasmic reticulum (ER/SR) is phosphorylated by CamKII on S2815, which results in Ca^2 +^ release from the ER/SR (Wehrens et al., 2004). To demonstrate that the Ryr Ca^2 +^-release channel is required for SRT1720 to activate AMPK, we treated cells with SRT1720 in the presence of the Ryr channel inhibitor ryanodine. As shown in Fig. 3I, ryanodine inhibited SRT1720 from activating AMPK. Then, to examine whether SRT1720 induced Ryr2 phosphorylation by CamKII, we treated C2C12 myotubes with SRT1720 in the presence of siRNA specific for Epac1. Immunoblotting with antibody specific for phospho-S2815 indicates that SRT1720 induces Ryr2 phosphorylation in an Epac1-dependent manner (Fig. 3J). To demonstrate that Ca^2 +^ signaling is important for SRT1720-mediated activation of AMPK, we treated the myotubes with SRT1720 in the presence of the calcium chelator BAPTA and found that BAPTA decreased SRT1720-induced AMPK activation (Fig. 3K). In order to examine whether SRT1720-mediated activation of Sirt1 is downstream of Epac, we treated C2C12 myotubes with SRT1720 in the presence of Epac1 siRNA or control siRNA. As shown in Fig. 3L, deacetylation of PGC-1α was diminished with Epac1 siRNA, indicating that activation of Sirt1 is, at least partially, downstream of Epac.

### 3.4 SRT1720 does not Increase Mitochondrial Content or Glucose Tolerance in AMPKα2 KO Mice

**Fig. 4:**
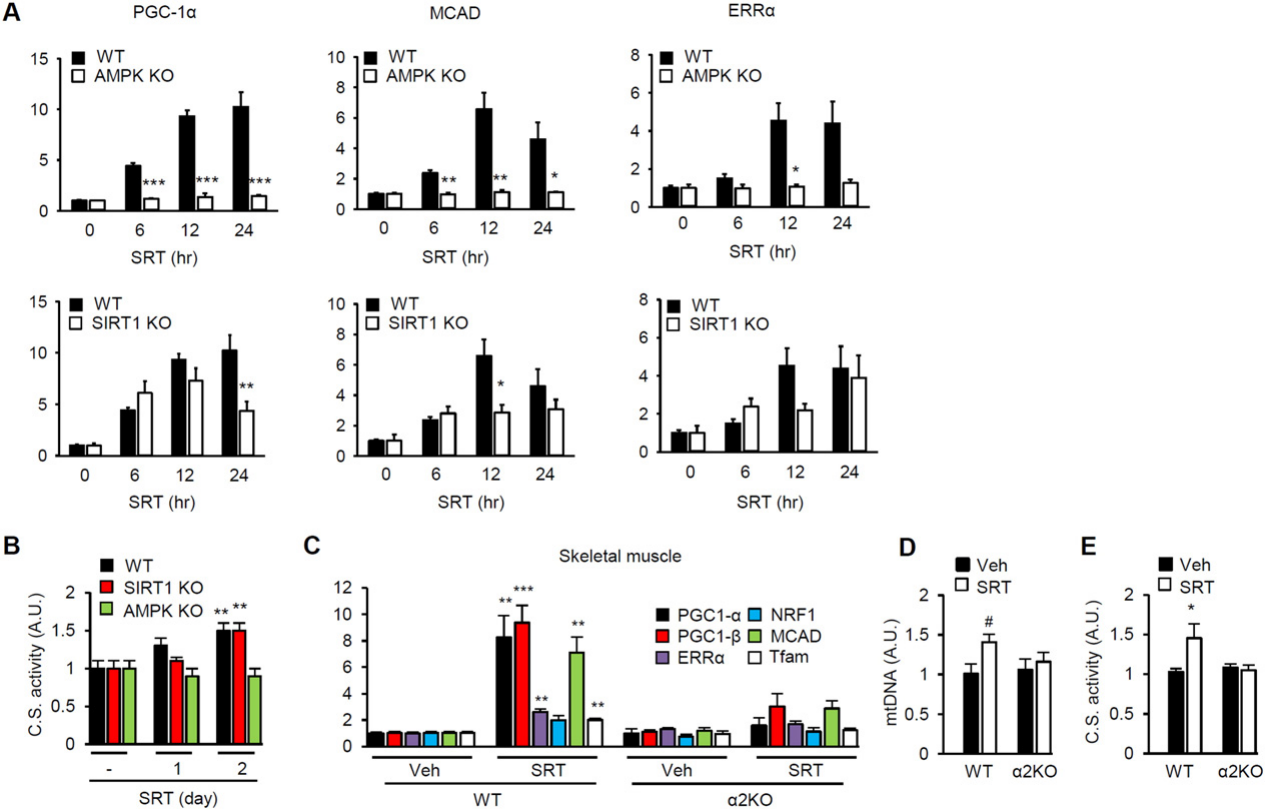
SRT1720-induced mitochondrial biogenesis requires AMPK. (A) Real-time PCR measurements of the mRNA levels of PGC-1α, MCAD and ERRα in WT, AMPKα1/α2 KO and Sirt1 KO mefs after treatment with SRT1720 for the indicated times. The mRNA levels at time 0 were arbitrarily set to one in all cases. All values are given as mean ± s.e.m. *p < 0.05; **p < 0.01; ***p < 0.001. (B) Citrate synthase activity was measured in WT, AMPKα1/α2 KO and Sirt1 KO mefs after treatment with SRT1720 for the indicated times. All values are given as mean ± s.e.m. **p < 0.01. (C) Skeletal muscle was isolated from WT and AMPKα2 KO mice treated with either vehicle or SRT1720 for 17 weeks. The mRNA levels (in arbitrary units) of genes important for mitochondrial biogenesis were measured by using real-time PCR (n = 5–6). All values are given as mean ± s.e.m. **p < 0.01; ***p < 0.001. (D) Relative mtDNA levels in skeletal muscle of WT and AMPKα2 knockout mice treated with either vehicle or SRT1720 (n = 5–7 per genotype). #p = 0.067. All values are given as mean ± s.e.m. (E) Citrate synthase activity was measured in skeletal muscle of WT and AMPKα2 knockout mice treated with either vehicle or SRT1720 (n = 5–7 per genotype). All values are given as mean ± s.e.m. *p < 0.05.

In order to investigate the role of AMPK in SRT1720-induced mitochondrial biogenesis, we treated mouse embryo fibroblasts (mefs) missing both α subunits (α1 and α2) of AMPK (AMPK KO). As shown in Fig. 4A, SRT1720 robustly induced the expression of mitochondrial biogenesis genes in WT but failed to induce them in AMPK KO mefs. Since SRT1720 is a Sirt1 activator, we then examined the mitochondrial biogenesis genes in Sirt1 KO mefs. We found that in Sirt1 KO mefs, all three genes were partially induced but the expression started to decline early (PGC-1α) or was induced more slowly (MCAD and ERRα) than in WT mefs (Fig. 4A). Consistent with this, the induction of mitochondrial citrate synthase activity was absent in AMPK KO mefs and slightly delayed in Sirt1 KO mefs (Fig. 4B). Taken together, these findings indicate that SRT1720 induces the expression of genes important for mitochondrial biogenesis in an AMPK- and partially in a Sirt1-dependent manner. Consistent with this, SRT1720 did not increase the expression of genes important for mitochondrial biogenesis (Fig. 4C), the mitochondrial content (Fig. 4D) or citrate synthase activity (Fig. 4E) in skeletal muscle of AMPKα2 KO mice. Taken together, these findings indicate that AMPK is absolutely required for SRT1720 activity on mitochondrial biogenesis.

**Fig. 5:**
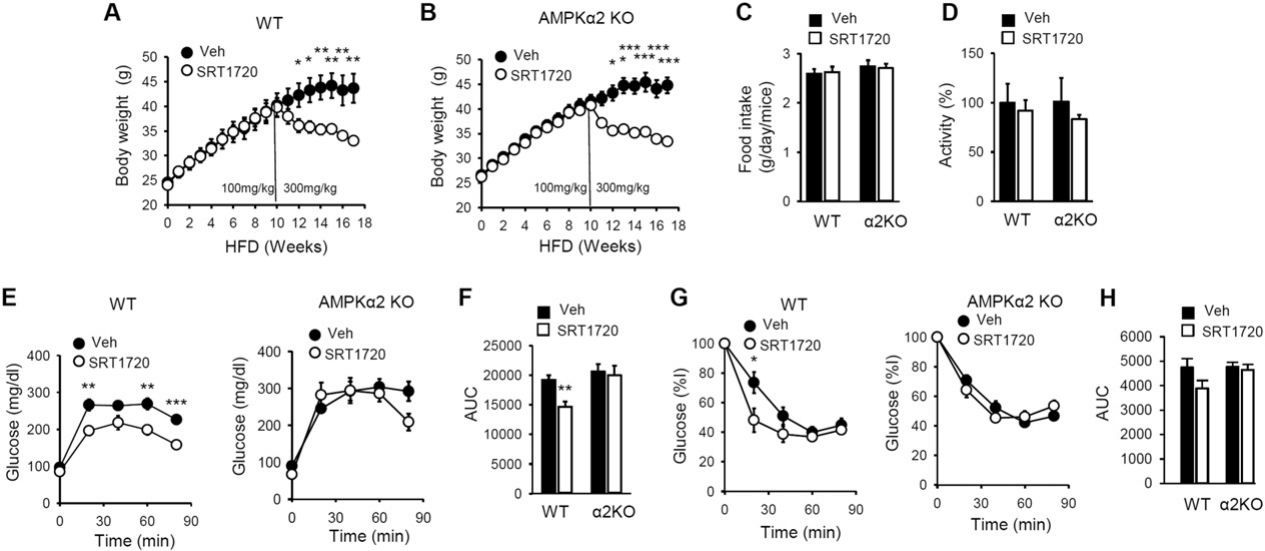
SRT1720 does not improve glucose homeostasis in AMPKα2 KO mice. Body weight of WT (A) and AMPKα2 KO (B) mice fed HFD (± SRT1720) (n = 5–7 per treatment group). All values are expressed as mean ± s.e.m. *p < 0.05; **p < 0.01; ***p < 0.001 (C) Food intake for WT and AMPK α2KO mice treated either vehicle or SRT1720 (n = 5–7 per each genotype). All values are expressed as mean ± s.e.m. (D) Relative locomotor activity for WT and AMPK α2KO mice fed either vehicle or SRT1720 (n = 5–7 per each genotype). All values are expressed as mean ± s.e.m. (E) Glucose tolerance of WT and AMPK α2KO mice on HFD (SRT1720) for 17 weeks are shown (n = 5–7 per treatment group). All values are given as mean ± s.e.m. **p < 0.01; ***p < 0.001. (F) Area under the curve (AUC) data for glucose tolerance of WT and AMPK α2KO mice on HFD (SRT1720) for 17 weeks are shown (n = 5–7 per treatment group). All values are given as mean ± s.e.m. **p < 0.01. (G) Insulin sensitivity of WT and AMPK α2KO mice on HFD (SRT1720) for 17 weeks are shown (n = 5–7 per treatment group). All values are given as mean ± s.e.m. *p < 0.05. (H) Area under the curve (AUC) data for insulin sensitivity of WT and AMPK α2KO mice on HFD (SRT1720) for 17 weeks are shown (n = 5–7 per treatment group). All values are given as mean ± s.e.m.

Previously, Feige et al. showed that SRT1720 (500 mg/kg/day) protected against diet-induced obesity and glucose intolerance (Feige et al., 2008). In order to examine the role of AMPK in the anti-diabetic effect of SRT1720, we repeated these experiments with WT and AMPKα2 KO mice by feeding them HFD containing SRT1720 (300 mg/kg/day). We escalated the dose to 300 mg/kg after mice were first adapted to a 100 mg/kg dose (Fig. 5A and B). Both WT mice and AMPKα2 KO mice lost weight equally when dosed with 300 mg/kg SRT1720 (Fig. 5A and B), even though SRT1720 did not affect food intake (Fig. 5C) or activity level (Fig. 5D) in either WT or AMPKα2 KO mice. We then performed a glucose tolerance test (GTT) (Fig. 5E) and calculated the area under the curve (AUC) (Fig. 5F), which showed that SRT1720 improved glucose tolerance in WT mice but not in AMPKα2 KO mice. Similarly, an insulin tolerance test (ITT) (Fig. 5G and H) indicated that SRT1720 increased insulin sensitivity in WT mice but not in AMPKα2 KO mice. Therefore, the anti-diabetic effects of SRT1720 require AMPK and in the absence of AMPKα2, weight loss induced by SRT1720 alone is not sufficient to improve glucose homeostasis.

## 4 Discussion

The metabolic effects of SRT1720 have been extensively studied (Feige et al., 2008, Milne et al., 2007, Minor et al., 2011a, Mitchell et al., 2014), but the mechanism behind its effect on glucose homeostasis is poorly understood. STACs were discovered as Sirt1 activators in a screen that utilizes a fluorophore-tagged substrate. However, a number of groups observed that STACs did not activate Sirt1 if the substrate peptide was not tagged with a fluorophore (Beher et al., 2009, Borra et al., 2005, Dai et al., 2010, Hubbard et al., 2013, Kaeberlein et al., 2005, Pacholec et al., 2010). It was reported that STACs can activate Sirt1 against native substrates that have a bulky hydrophobic amino acid residue at either position + 1 or + 6 (Dai et al., 2015, Dai et al., 2010, Hubbard et al., 2013), but this is still controversial (Cao et al., 2015). Either way, this means that STACs cannot directly activate Sirt1 activity against the vast majority of the endogenous substrates. Indeed, out of 13 lysine residues in the Sirt1 substrate PGC-1α, only one lysine (K 778) fulfilled the hydrophobic residue criteria, and we found that mutating K778 to Q still allowed deacetylation of the remaining lysines of PGC-1α after SRT1720 treatment (Fig. 1A). Nevertheless, the metabolic effects of SRT1720 are impaired in conditional Sirt1 KO mice (Minor et al., 2011a, Mitchell et al., 2014).

The Sirt1 requirement for SRT1720 action (Feige et al., 2008, Minor et al., 2011b, Mitchell et al., 2014) does not necessarily mean that SRT1720 mediates these actions by activating Sirt1; it is possible that basal activity of Sirt1 may be required and is sufficient for these effects. In fact, unlike SRT1720 treatment (Feige et al., 2008), Sirt1 gain of function does not mimic CR nor increase mitochondrial function in skeletal muscle (Boutant et al., 2016). If SRT1720 acts solely by activating Sirt1, then the salient effects of SRT1720 such as the stimulation of expression of mitochondrial genes (*e.g.* PGC-1α) should not occur in Sirt1-deficient cells. However, partial and muted response (6–12 h) to SRT1720 is present in Sirt1-deficient mefs (Fig. 4A), suggesting that another pathway is also required for the full effect of SRT1720. Indeed, SRT1720 activates AMPK in a Sirt1-independent manner, and AMPK is absolutely required for the metabolic effects of SRT1720 both in mefs and in obese mice (Fig. 4, Fig. 5). Our results contradict previous studies that reported SRT1720 does not acutely activate AMPK in C2C12 myotubes (Feige et al., 2008, Svensson et al., 2015). However, this discrepancy is most likely due to using different SRT1720 concentrations as well as visualizing AMPK activity at different time points (Figs. 1B and C).

It should be noted that the concentration of SRT1720 used for i.p. injection (Fig. 1D, F) is considerably lower than that added to the food (Fig. 5): 10–30 mg/kg *vs.* 300 mg/kg/day. The reason for this is that the dose given i.p. is delivered nearly instantaneously whereas the dose in the food is consumed over a 24 h period.

SRT1720 leads to weight loss in obese mice (Feige et al., 2008), but the effect of weight loss on glucose homeostasis has not been investigated. Our finding that SRT1720 decreases body weight equally in both WT and AMPKα2 KO mice, but does not increase glucose tolerance or mitochondrial content in AMPKα2 KO mice indicates that the effect of SRT1720 on glucose homeostasis is not simply due to weight loss (Fig. 5). The reason weight loss occurred in AMPKα2 KO mice is most likely due to the fact that AMPKα1, not AMPKα2, is the dominant AMPK in adipose tissue (Daval et al., 2005, Lihn et al., 2004). Consistent with this, resveratrol reduced body weight in AMPKα2 KO mice but not in AMPKα1 KO mice (Um et al., 2010). Viollet et al. reported that untreated AMPKα2 KO mice were less glucose tolerant than untreated WT mice, which conflicts with our findings (Fig. 5) (Viollet et al., 2003). The most likely reason for this difference is that Viollet et al. measured glucose tolerance with mice on regular chow, whereas we measured glucose tolerance with mice which were made obese by feeding HFD; the HFD and obesity it induced could have masked any difference in glucose tolerance without treatment.

**Fig. 6:**
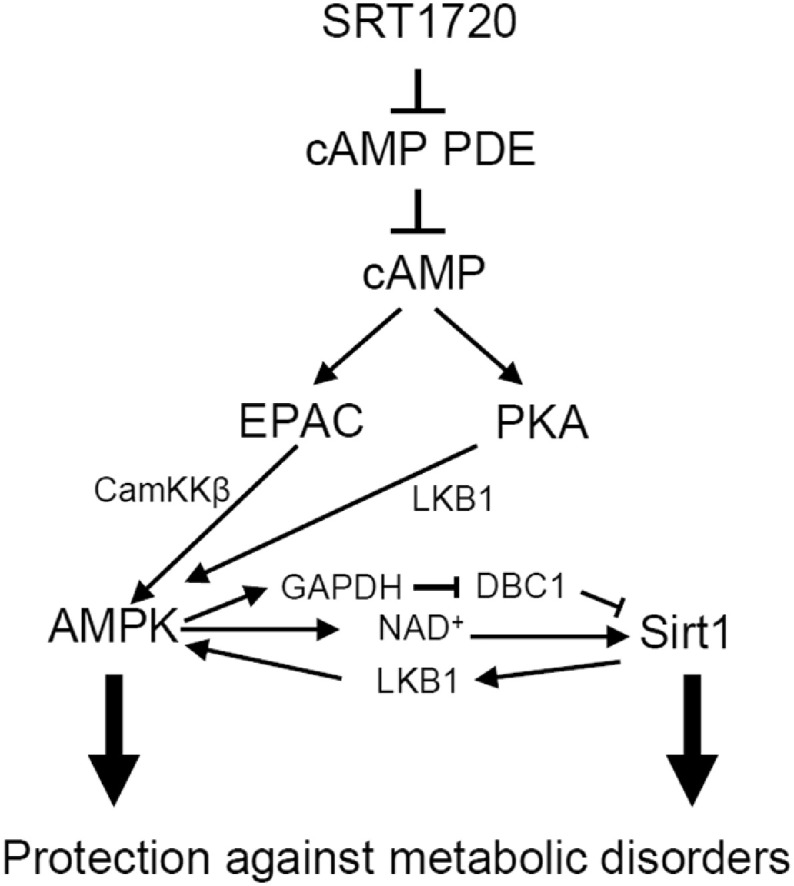
Schematic diagram of SRT1720 action. SRT1720 improves glucose homeostasis and protects against metabolic disorders by activating AMPK and Sirt1 *via* interlinked pathways. Although we were not able to confirm the role of the PKA-LKB1 pathway in SRT1720 effect, the PKA-LKB1 link has been reported previously (Shelly et al., 2007).

Our results indicate that SRT1720, like resveratrol (Park et al., 2012), increases AMPK activity and cAMP signaling by inhibiting cAMP PDEs. Pacholec et al. also found that PDEs were inhibited by resveratrol and the SRT compounds, including SRT1720 (Pacholec et al., 2010). It appears that activation of Sirt1 by SRT1720 is downstream of Epac/AMPK (Fig. 3L). Therefore, our findings, together with previous studies (Feige et al., 2008, Minor et al., 2011a, Mitchell et al., 2014), indicate that the metabolic effects of SRT1720 are mediated by both AMPK and Sirt1, which are activated by independent pathways (Fig. 6). The effects of cAMP on AMPK and Sirt1 activities are complicated: it has been reported that Sirt1 can be activated by cAMP directly (Wang et al., 2015) as well as by a kinase(s) downstream of cAMP (Gerhart-Hines et al., 2011, Nin et al., 2012) and PKA can activate LKB1, an upstream kinase for AMPK (Shelly et al., 2007). Which of these pathways is dominant most likely depends on the cell types and the physiological context. Although the physiological effects of cAMP are diverse, there are evidences of beneficial effects of cAMP signaling in amelioration of the aging-related phenotype. For example, exogenous cAMP increases lifespan in fruit flies (Tong et al., 2007) and activates AMPK and Sirt1 and mimics the anti-aging effects of calorie-restriction in mice (Wang et al., 2015). Increasing the cAMP level with the PDE4 inhibitor rolipram (Park et al., 2012) or roflumilast (Tikoo et al., 2014, Wouters et al., 2012) also activates AMPK and Sirt1 and protects against obesity and type 2 diabetes in mice and humans. In addition, Sirt1 expression level decreases with aging, but this is rescued by cAMP (Wang et al., 2015).

The increase in total cAMP level after SRT1720 appears modest (~ 20–70% at 0.2–2.5 μM SRT1720), but this is most likely an underestimation because cAMP exists in compartments (Mongillo et al., 2004); the cAMP level in the relevant compartments could be significantly higher than in the rest of the cell. Hubbard et al. reported that STACs, including resveratrol did not inhibit PDEs (Hubbard et al., 2013), which contradicts our finding and that of Pacholec et al. (Pacholec et al., 2010). The most likely explanation for this is that because SRT1720 and resveratrol are competitive cAMP PDE inhibitors their inhibitory activity depends on the concentration of cAMP used in the PDE assay. We used 10 nM of cAMP, a concentration commonly used in PDE inhibition assays (Manganiello and Vaughan, 1973). Indeed, we also found that SRT1720 and resveratrol have no activity in the PDE inhibition assay (data not shown) if we used cAMP in the micromolar range (Hubbard et al., 2013). However, at this concentration, even the PDE4 inhibitor rolipram does not have significant activity in the PDE inhibition assay (Fig. 2G).

It is intriguing that so many compounds reported to be Sirt1 activators are also PDE inhibitors. In addition to the SRT compounds and resveratrol (Pacholec et al., 2010, Park et al., 2012), other compounds that were reported to be Sirt1 activators such as butein, fisetin and quercetin (Howitz et al., 2003) have also been shown to inhibit recombinant PDEs (Kuppusamy and Das, 1992). Since they were all identified by an assay that uses the fluorophore-tagged peptide as a Sirt1 substrate, it is tempting to speculate that this assay, for reasons we do not understand, has a tendency to select compounds that can also fit into the catalytic pocket of cAMP PDEs.

Currently, SRT1720 is widely used and accepted as a Sirt1-specific activator. This work shows that the mechanism of its action, particularly in regards to its metabolic effects may occur not solely through Sirt1 but also through the cAMP and AMPK pathways. Therefore, it raises the possibility that some conclusions drawn with experiments using STACs such as SRT1720 may need to be reevaluated.

Another strategy to directly activate Sirt1 is to increase the intracellular concentration of its cofactor NAD^+^. Indeed, treatment with NAD^+^ precursors such as nicotinamide mononucleotide or nicotinamide ribose increase intracellular NAD^+^ levels and confer metabolic benefits (Canto et al., 2012, Gomes et al., 2013, Mouchiroud et al., 2013, Pirinen et al., 2014, Yoshino et al., 2011). Although the effects of the NAD^+^ precursors have been attributed to activation of sirtuins, it has been reported that NAD^+^ also directly activates AMPK with a K_m_ that is comparable to that for Sirt1 (Rafaeloff-Phail et al., 2004). Therefore, it would be interesting to examine what role, if any, AMPK plays in the physiological effects produced by increasing intracellular NAD^+^ levels.

## Funding Sources

This work was supported by the Intramural Research Program, National Heart Lung and Blood Institute (HL006119-06) and the National Institute of Arthritis and Musculoskeletal and Skin Diseases (AR041164-17), National Institutes of Health.

## Author Contributions

S.J.P. performed the majority of experiments and analyzed the data and wrote the paper, F.A. performed the *in vitro* study with PDEs1-5. J.H.U., A.L.B., X.X. and H.K. helped with experiments. H.K. performed docking of SRT1720 to the PDE3B active site. J.R., X.F. and V.S. generated muscle specific Sirt1KO mice. A.P. and S.S. helped during experiments. M.K.K. and J.H.C. designed and supervised the study, analyzed the data and wrote the manuscript.

## Conflicts of Interest

The authors declare that they have no conflicts of interest with the contents of this article.
